# Tumors of Atypical Carcinoma of the Parotid Gland and Papillary Thyroid Carcinoma: A Case Report

**DOI:** 10.7759/cureus.10496

**Published:** 2020-09-16

**Authors:** Aileen Kerns, Andrew Ross, Eric M Sugihara, Samba Siva Bathula

**Affiliations:** 1 Department of Otolaryngology–Head and Neck Surgery, Detroit Medical Center, Detroit, USA; 2 Department of Otolaryngology–Head and Neck Surgery, Detroit Medical Center/Michigan State University, Detroit, USA

**Keywords:** lymphoepithelial carcinoma, facial nerve paralysis, papillary thyroid carcinoma, parotid gland, salivary gland malignancy, thyroid cancer

## Abstract

Lymphoepithelial carcinoma (LEC) is a variant of anaplastic carcinoma usually found in the nasopharynx. It is a rare, aggressive malignant tumor in the salivary glands, which is usually associated with Epstein-Barr virus (EBV), and often presents with facial nerve paralysis when in the parotid gland. This case report is unique in that our patient had EBV-negative LEC, with facial nerve involvement and a concurrent primary papillary thyroid carcinoma (PTC). We successfully managed this patient with surgery and adjuvant chemoradiotherapy. The patient has responded well to the treatment and she showed no evidence of disease at the 24-month follow-up.

## Introduction

Lymphoepithelial carcinoma (LEC) is a variant of anaplastic carcinoma usually found in the nasopharynx and rarely in salivary glands [1]. This tumor is most often associated with Epstein-Barr virus (EBV) in endemic areas [2]. The first case of LEC in a salivary gland was described by Hilderman et al. in 1962 and accounts for approximately 0.3-6% of malignant salivary gland tumors [3]. LEC usually presents with parotid swelling and concurrent cervical adenopathy. It is often painful and typically presents with facial nerve paralysis [4]. The occurrence of LEC in the parotid gland with facial nerve paralysis along with co-primary of papillary thyroid carcinoma (PTC) is very unique [5]. We present the case of an African American female with EBV-negative LEC of the parotid gland with complete facial nerve paralysis and a concurrent primary PTC. To the best of our knowledge, this is the first described case of EBV-negative LEC with concurrent primary PTC in the English-language literature.

## Case presentation

A 50-year-old African American woman presented to our otolaryngology clinic for a right parotid mass that she had first noticed two months prior. She had associated right facial pain, weakness, numbness, and dry eye. She had a history of hypertension managed with amlodipine. She had no family history of cancer. She was a non-smoker and denied alcohol and illicit drug use. On physical examination, she had a 4 x 3cm right parotid mass that was fixed and tender. She had right facial nerve paralysis of all divisions consistent with House-Brackmann grade VI function without palpable cervical lymphadenopathy. Clear saliva was expressed from Stenson’s duct on the tumor side. The remainder of the head and neck exam was unremarkable.

**Figure 1:**
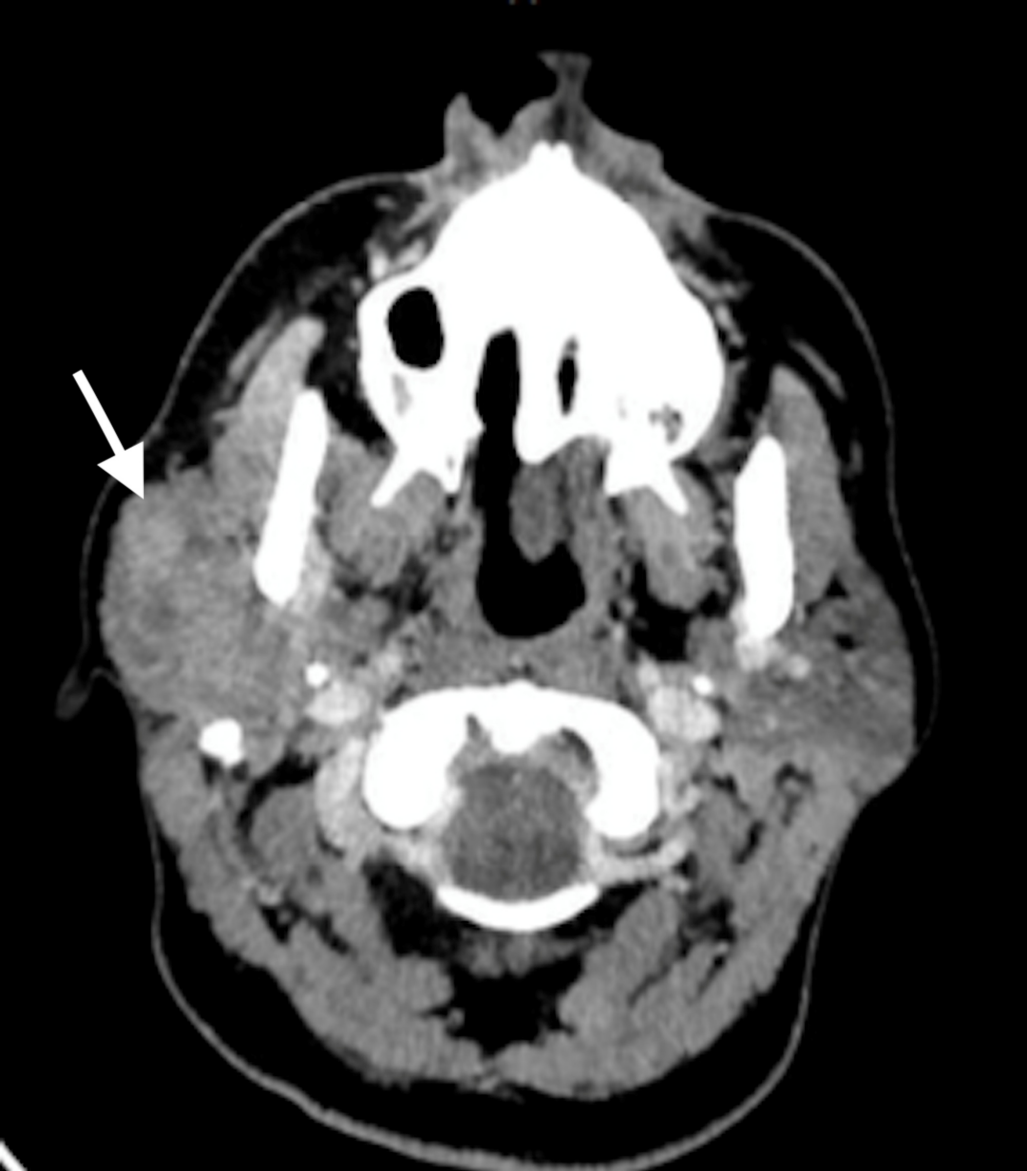
Contrasted CT neck image in axial view The image shows an infiltrating heterogeneously enhancing mass of the superficial and deep lobes of the right parotid gland with cervical lymphadenopathy of level 2 (arrow) CT: computed tomography

A contrasted CT of the neck study was obtained and showed a poorly defined, heterogeneously enhancing, infiltrative mass involving the superficial and deep lobes of the right parotid gland (Figure 1).

**Figure 2:**
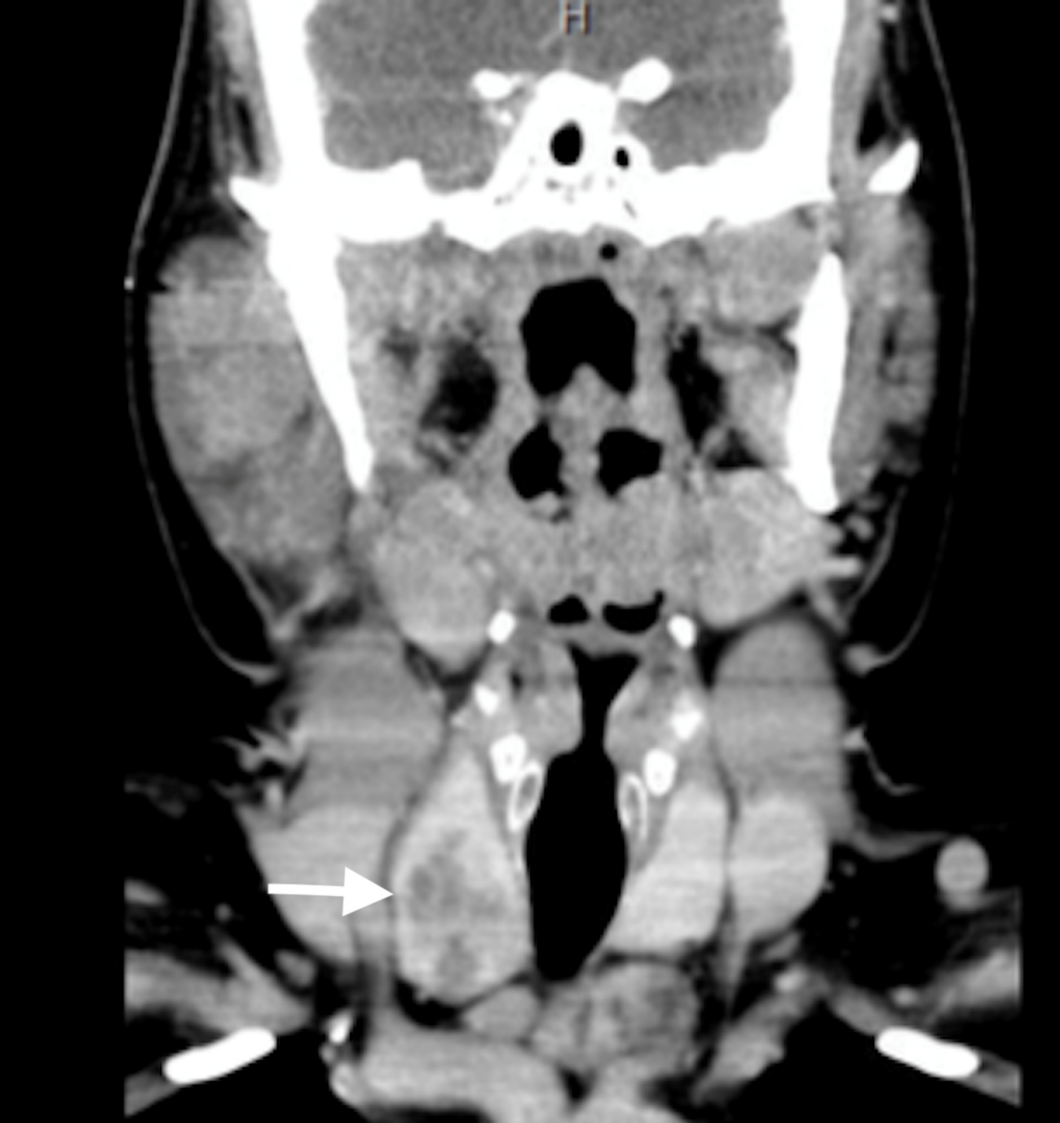
Contrasted CT neck image in the coronal plane The image shows incidental calcifications in the right thyroid lobe (arrow) CT: computed tomography

There was no evidence of perineural spread as the stylomastoid foramen did not appear enlarged. There were slightly enlarged cervical lymph nodes involving levels Ib, II, and V, with the largest lymph node measuring 1.7 x 1.3cm. In addition, a heterogeneously enhancing, cystic mass in the right lobe of the thyroid measuring 2.5 x 3.4 x 3.0cm and a mass lying inferior to the left lobe of the thyroid measuring 4.4 x 2.8 x 2.7cm were incidentally found (Figure 2). A CT thorax showed no metastatic disease.

A right-sided radical parotidectomy was performed en bloc with the facial nerve due to gross infiltration and absent stimulation of all divisions except the cervical branch. Frozen section specimen analysis confirmed malignancy intraoperatively. An ipsilateral selective neck dissection was performed involving levels Ib through V with the preservation of the spinal accessory nerve, internal jugular vein, and sternocleidomastoid muscle. In addition, a right hemithyroidectomy and an intrathoracic thyroidectomy were performed.

**Figure 3:**
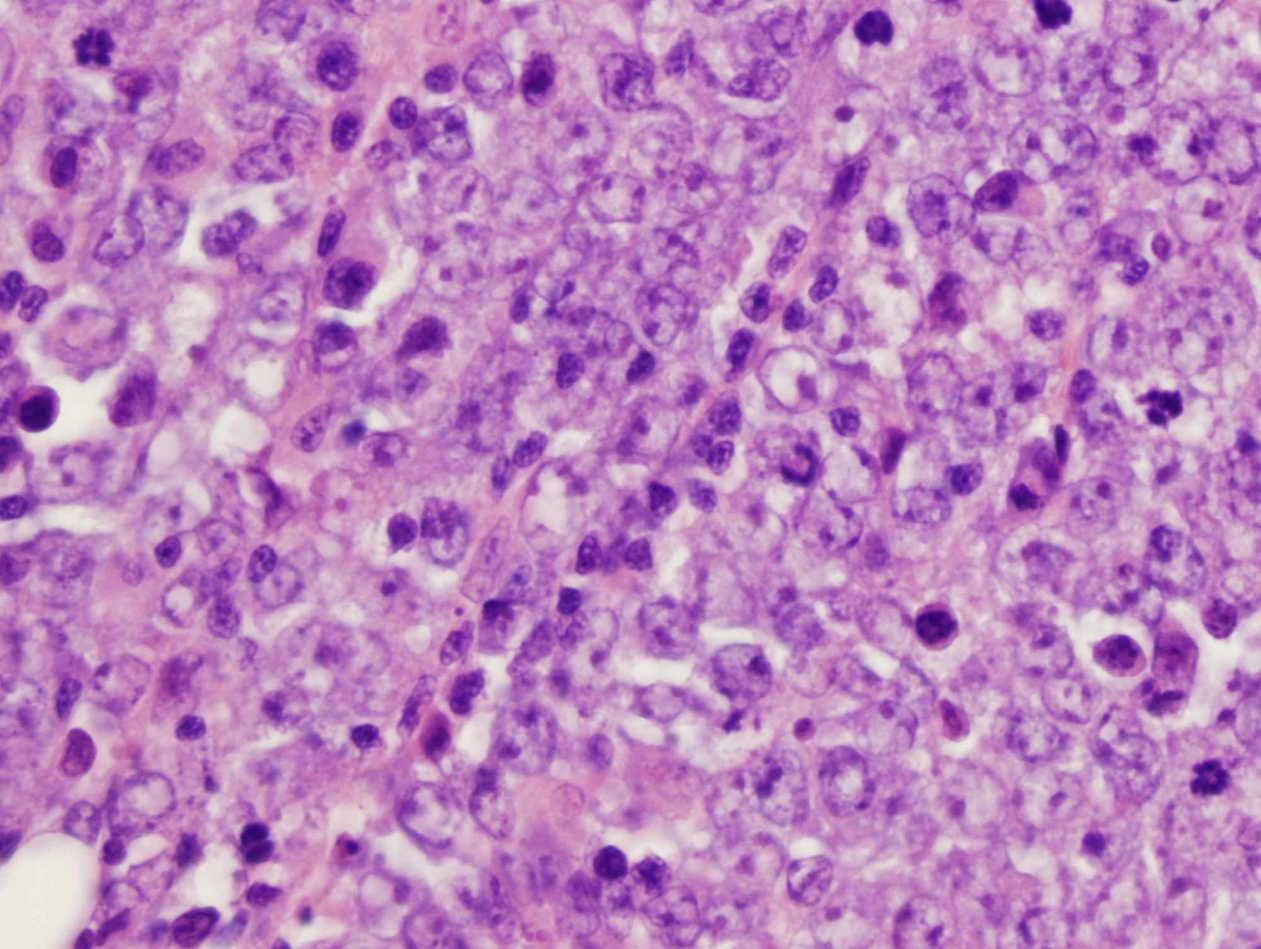
H&E stained slide The slide shows a diffuse tumor growth pattern with sheets of malignant cells and large vesicular nuclei with prominent nucleoli. There are some lymphocytes present (400X) H&E: hematoxylin & eosin

Pathology showed a poorly differentiated carcinoma of the right parotid gland with extraparenchymal extension. The neoplasm involved the lymphoid stroma with sheets of epithelioid cells and pleomorphism, the histologic patterns and staining were consistent with LEC (Figure 3).

**Figure 4:**
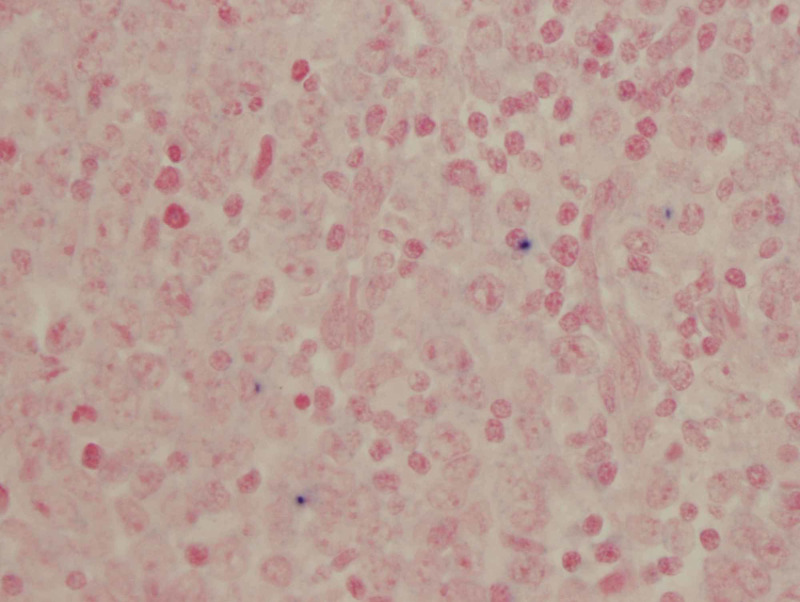
Immunohistochemical staining Tumor cells negative (no stain) for EBV-encoded RNA by in-situ hybridization (400X). Positive studies show darkly stained nuclei of EBV-infected cells EBV: Epstein-Barr virus; RNA: ribonucleic acid

Infiltration of the facial nerve trunk was confirmed, and the tumor margins were negative. Immunohistochemical staining was performed, which was negative for Epstein-Barr-encoded RNA (Figure 4).

Metastatic disease was identified in one lymph node in level IIA and two lymph nodes in IIB. The parotid disease was staged T4aN2bM0 (IVA). The right thyroid mass was found to be of the follicular variant of primary PTC and the intrathoracic mass was a nodular hyperplasia. The thyroid disease was staged pT2N0M0(I).

The patient received adjuvant and concomitant chemoradiotherapy with high-dose cisplatin and 64 Gray of intensity-modulated radiation therapy (IMRT) to the right side of the neck and face. Following 24 months after completion of the therapy, the positron emission tomography-CT (PET-CT) showed no evidence of disease in the parotid. Since the PTC was fully removed and there was no evidence of lymph node or extrathyroidal spread, radioactive iodine was not indicated for this patient. The complete facial paralysis was managed with gold weight placement. She has recovered well and has shown no clinical or radiographic evidence of disease 24 months after surgery.

## Discussion

LEC is a rare aggressive malignant tumor in the parotid gland. Individuals of Native American, Southeastern Chinese, and Japanese descent are at greater risk of this condition. It has an association with the EBV [6]. The histopathological characteristics that make this tumor unique is a prominent lymphoid stroma. The pathological description for LEC remains similar across the reported cases, with the tumor usually found in the nasopharynx and rarely in the salivary glands, affirming our findings of a LEC involving our patient’s parotid gland.

Ambrosio et al. completed a Western literature review where they found that six out of the nine cases that reported EBV status were positive for LEC [6]. In addition, they reported one patient with unknown EBV status who had a neural invasion. Also, Wang et al. found that all 16 cases in their review were EBV-positive and reported one patient who had preoperative, progressive facial nerve palsy [7]. Our patient had preoperative facial nerve paralysis, tumor invasion of the facial nerve, and was EBV-negative. EBV appears to be a risk factor for the development of LEC in endemic populations, but its effect on tumor biology in aggressive disease is unclear. Due to high radiosensitivity, the treatment of choice for LEC is surgery with adjuvant radiotherapy, and this combination method has shown improved survival outcomes over surgery alone [8].

LEC with a co-primary of PTC is extremely rare, and only one case has been reported, by Bishnoi et al., in the literature that was EBV-positive LEC [5]. But our present case report is unique and will contribute immensely to current literature as it involves a rare case of EBV-negative status with a co-primary of PTC in an African American female. However, it is unclear if EBV status portends a more aggressive malignant disease status.

## Conclusions

LEC is very aggressive in the parotid gland and is often associated with facial nerve paralysis and cervical lymphadenopathy. Here we presented an unusual case of LEC of the parotid gland with characteristic facial nerve paralysis, but EBV negativity associated with a concurrent primary PTC. To the best of our knowledge, this is the first described case of a patient with EBV-negative LEC of the parotid gland with facial nerve invasion and concurrent, primary PTC. The patient was successfully managed with surgery and adjuvant radiotherapy, showing no evidence of disease at the 24-month follow-up.

## References

[1] Schneider M, Rizzardi C (2008). Lymphoepithelial carcinoma of the parotid glands and its relationship with benign lymphoepithelial lesions. Arch Pathol Lab Med.

[2] Lanier AP, Bornkamm GW, Henle W, Henle G, Bender TR, Talbot ML, Dohan PH (1981). Association of Epstein-Barr virus with nasopharyngeal carcinoma in Alaskan native patients: serum antibodies and tissue EBNA and DNA. Int J Cancer.

[3] Ban X, Wu J, Mo Y, Yang Q, Liu X, Xie C, Zhang R (2014). Lymphoepithelial carcinoma of the salivary gland: morphologic patterns and imaging features on CT and MRI. AJNR Am J Neuroradiol.

[4] Wenig B, Heffess C (2008). Neoplasms of the salivary glands: lymphoepithelial carcinoma. Atlas of Head and Neck Surgery.

[5] Bishnoi R, Kaushal S, Varma MC, Shukla NK, Ray R (2010). Double primary-lymphoepithelioma-like carcinoma of the parotid and papillary carcinoma of the thyroid. Indian J Cancer.

[6] Ambrosio MR, Mastrogiulio MG, Barone A (2013). Lymphoepithelial-like carcinoma of the parotid gland: a case report and a brief review of the western literature. Diagn Pathol.

[7] Wang CP, Chang YL, Ko JY, Lou PJ, Yeh CF, Sheen TS (2004). Lymphoepithelial carcinoma versus large cell undifferentiated carcinoma of the major salivary glands. Cancer.

[8] Abdulla AK, Mian MY (1996). Lymphoepithelial carcinoma of salivary glands. Head Neck.

